# The legal obligation to provide timely security patching and automatic updates

**DOI:** 10.1365/s43439-022-00059-6

**Published:** 2022-09-06

**Authors:** Alana Maurushat, Kathy Nguyen

**Affiliations:** 1 Western Sydney University, Sydney, Australia; 2 CyberCX, Brisbane, Australia

**Keywords:** Cybersecurity, Cybersecurity compliance, Cyber risk management, Corporate responsibility, Vulnerability and disclosure, Cybersicherheit, Einhaltung von Cybersicherheitsvorschriften, Cyberrisikomanagement, Unternehmensverantwortung, Schwachstellen und Offenlegung

## Abstract

Do you use Office 365 or Windows 10? How about GoDaddy to support your website? Has it been a while since you connected your iPhone to Wi-Fi instead of merely running off your data? Or is your Samsung phone more than 2 years old? Would it surprise you to learn that some of these products no longer receive security support or automatic updates? If so, you may be surprised to hear that you are being exposed to security risks, as many cyber incidences are the direct result of an absence of security patching and automatic updates. There are many reasons for this. Most companies provide security patches, but they are not always timely and many are not automated, requiring manual effort (often unbeknownst to consumers and businesses). Timely security patching is, upon discovery or notification of a security flaw in a system or product, the release of a security update within a reasonable time that patches and updates the security of a system—sometimes this is automatic, sometimes the security patch is merely a notification that you can and should patch your own system. A contributing factor to this is that there is no legal obligation to provide security support, let alone timely security support. This means that there is no legal requirement to patch known security vulnerabilities and bugs or issue automatic updates. This paper asks whether or not Australia should have a legal obligation to ensure timely security patching and require automatic updates by default in all consumer systems. Our conclusion: yes, it should, since many companies cannot be relied on to self-regulate and put their client’s security interests first, and the stakes in cybersecurity have become too high to continue with the status quo. We conclude by presenting our recommended pathway for legal reform.

## Abstract (de)

Verwenden Sie Office 365 oder Windows 10? Wie ist es mit GoDaddy zur Unterstützung Ihrer Website? Ist es schon eine Weile her, dass Sie Ihr iPhone mit WLAN verbunden haben, statt es nur mit Ihren mobilen Daten zu betreiben? Oder verwenden Sie ein mehr als zwei Jahre altes Samsung-Telefon? Würde es Sie überraschen zu erfahren, dass einige dieser Produkte keine Sicherheitsunterstützung oder automatischen Updates mehr erhalten? Wenn ja, überrascht es Sie vielleicht, dass Sie dadurch Sicherheitsrisiken ausgesetzt sind, denn viele Cybervorfälle sind das direkte Ergebnis fehlender Sicherheitspatches und automatischer Updates. Dafür gibt es viele Gründe. Die meisten Unternehmen stellen zwar Sicherheitspatches zur Verfügung, aber nicht immer rechtzeitig, und viele davon sind nicht automatisiert und erfordern manuellen Aufwand (oft ohne Wissen der Verbraucher und Unternehmen). Rechtzeitige Sicherheitspatches bedeuten, dass nach der Entdeckung oder Meldung einer Sicherheitslücke in einem System oder Produkt innerhalb eines angemessenen Zeitrahmens ein Sicherheitsupdate veröffentlicht wird, das die Sicherheit des Systems verbessert und aktualisiert – manchmal geschieht dies automatisch, manchmal ist der Sicherheitspatch eine bloße Benachrichtigung, dass Sie Ihr eigenes System patchen können und sollten. Dazu trägt auch bei, dass es keine rechtliche Verpflichtung zur Sicherheitsunterstützung gibt, geschweige denn zur rechtzeitigen Sicherheitsunterstützung. Das bedeutet, dass es keine rechtliche Verpflichtung gibt, bekannte Sicherheitslücken zu schließen und Fehler zu beheben oder automatische Updates herauszugeben. In diesem Beitrag wird die Frage gestellt, ob es in Australien eine gesetzliche Verpflichtung geben sollte, Sicherheitslücken rechtzeitig zu schließen und automatische Updates für alle Verbrauchersysteme herauszugeben. Unsere Schlussfolgerung: Ja, das sollte es, denn man kann sich bei vielen Unternehmen nicht darauf verlassen, dass sie sich selbst regulieren und die Sicherheitsinteressen ihrer Kunden priorisieren. Zudem sind die Risiken im Bereich der Cybersicherheit zu hoch, um am Status quo festzuhalten. Abschließend stellen wir unseren empfohlenen Weg für eine Rechtsreform vor.

## Introduction

Here we are in 2022 with cyberwar imminently on our doorstep due to the Russian and Ukraine conflict, and yet we have known security vulnerabilities routinely used in cyber-attacks identified as early as 2019 that have still not be patched and continue to be exploited widely on a regular basis—so much so that they are on the list of Top Routinely Exploited Vulnerabilities, which is cybersecurity advice put out by the Joint Cybersecurity Advisory consisting of members from the U.S. Cybersecurity and Infrastructure Security Agency, the Australian Cyber Security Centre, the United Kingdom’s National Security Centre, and the U.S. Federal Bureau of Investigation (https://www.cisa.gov/uscert/ncas/alerts/aa21-209a).

One contributing factor why security vulnerabilities remain unpatched is that there is no legal requirement to continue security support of products or to issue timely security updates and patching. This is, naturally, a very complex ecosystem with many contributing factors including technical feasibility, economic motivation to fix, corporate ethics and others. Explaining how/why known security vulnerabilities remain unpatched and exploited for cybercrime and cyberwar would require a long and detailed book or television series.

This article has a simpler mission of convincing the reader that the era of no legal compliance for fixing software bugs and providing timely security patching needs to comes to an end. Self-regulation has not worked. If it had, we would not see security vulnerabilities from 2019 used on a daily basis for cyber-attacks and cybercrimes, most noticeably ransomware, payment diversion fraud and in the months to come, as a vector to cyberwar. The European Union is moving towards the legal obligation of timely security patching, and Australia should follow suit.

This paper will start by introducing essential terms, and briefly explore the underworld of how criminals purchase, discover and use security flaws to break into your systems and commit crimes such as ransomware, fraud, data breach, espionage and others. The second part looks more deeply why timely security support/patching coupled with the automatic updates by default are essential tools to combat cybersecurity threats. Part three and four examine the legal frameworks for security patching in Australia and the European Union. Part five concludes by providing possible legal reforms.

##  Why security patching?

What is a security patch and why is it needed? Before we can get to the many facets of this question, we need to know more about security vulnerabilities and bugs, why they are so prevalent and how they can be fixed along with the role that security patches and updates play in protecting our systems.

While there are many reasons for cyber threats, the main threats include cybersecurity breaches and cybercrimes for economic reasons, followed closely by political and social reasons. Most cyber-attacks occur to make money. These are perpetrated by organised criminal syndicates, terrorism finance, and rogue nation states whose economies now rely on the profits from cybercrime, mostly notably through scams, ransomware and payment diversion fraud. North Korea for example has fuelled an estimated $2 billion USD for weapons through cybersecurity breaches including ransomware and cryptocurrency hacking.^1^

Let’s take payment diversion fraud and ransomware for example. Payment Diversion Fraud (PDF) is a type of cyber-attack where an entity is tricked into making a direct payment from its account to a false supplier/entity often using real-time payment methods. Ransomware attack requires a vector for the covert deployment of an infection to the victim. How do criminals gain a foothold in your systems? They do so through vectors. Common vectors are: malicious emails/social engineering^2^, compromised credentials, brute force—remote desktop protocol^3^, exploit kits^4^, malware advertising^5^, drive-by-download^6^, and through security vulnerabilities.

While there are many forms of threat vectors, the most serious arise from exploiting *security vulnerabilities* as this can lead to system exploitation of every device and system globally that uses the technology. A security vulnerability is a software vulnerability or weakness in a computer system that can be exploited by an attacker. The vulnerability may exist as a concept or idea and need necessarily to be embodied in the software. An *exploit* is the implementation, in software, of a vulnerability. A *zero day exploit* is an exploit or vulnerability that is exploited against a target on the day on which public awareness of the existence of the vulnerability occurs—that is, zero days have elapsed between awareness and the use. Put into a physical context, imagine that you own a business in a physical building. You secure your business through locked doors, windows, keys, alarms, perhaps bio swipe cards and others. A security vulnerability could be that your door is only secured with what you think is a secure lock, but in fact the lock is easily disabled when you hold a magnet close to it coupled with a theft device (purchased on the Internet for $10). The exploit is the use of the magnet and theft device to break the lock. A *zero day vulnerability*, however, is a whole different thing because it is not common knowledge that there is a security vulnerability—neither the criminals, the manufacturers of the locks or the customers know about this type of vulnerability (which is why you can sell a zero day vulnerability from anywhere from $10,000 to over $1 million USD). The problem with software as opposed to physical locks is that they require updates and patching on a routine (if not daily) basis in order to make them secure.

Responsible organisations upon learning of a security vulnerability in their systems, issue what is known as a security patch and send out updates for users to install the latest security update. Sometimes these updates are automatically installed. Think Apple and iOS. But many require the end user to manually install the security patch. But there are too many security vulnerabilities—that have never been patched and are still in major use as the prime vector for cyber-attacks. These vulnerabilities are often in products of major technology companies that most organisations use to date. These known vulnerabilities are referred to as CVEs—common vulnerabilities and exposures—which are captured in the U.S. National Vulnerabilities Database. There are CVEs from 2018 in common products and software in Microsoft, Citrix, Fortinet, and more which remain common exploit vectors.^7^

Why are many of these systems still unpatched? There are many reasons. The first being the complexity of software. For example, the Apollo 11 spacecraft was launched and landed on the moon in 1969 with 145,000 lines of source code, a relatively recent version of a modern computer operating system, Apple’s OSX, comprises 86 million lines of code.^8^ It is estimated that Google controls a code base of over 2 billion lines of code.^9^ Yet there are more reasons than ‘it’s complicated’. Reasons vary, but one key reason is that the company may just decide to no longer support the product as it is more effective and economical to move a customer to a newer more secure product. Think everything Microsoft. While many companies do release security patches, not all of these patches are automatic. This means that the onus to implement that patch is on the user. In other words, they do not do automatic updates. Think some Android implementations in smart phones and many website platform companies.

Security patching and automatic updates are not easily navigable by users. Researchers at CACE (Centre for Cybersecurity Aid and Community Engagement) at Western Sydney University, conducted research where they analysed automatic update functionality in operating systems, browsers, emails and smart phones. The results are, frankly, terrifying and demonstrate the complexity and built-in assumptions in these systems. We have put the information into Table 1, commencing with an analysis of Windows 10, still the most common operating system in the world, especially for personal use and by small and microbusinesses with limited funds to update systems and software packages.

**Table 1:** Analysis of security patching for popular operating systems, browsers, email software and smart phones

**OPERATING SYSTEMS**	**Version**	**Supported/patched**	**Not Supported**
Windows 10	2004	X	–
	1909	X	–
	1903	X	–
	1809	X	–
	20H2	X	–
	1507	–	X
	1511	–	X
	1607	–	X
	1703	–	X
	1709	–	X
	1803	–	X
Windows 8	*All versions no longer supported as of January 14, 2020*	–	X
Windows 7	*All versions no longer supported as of January 14, 2020*	–	X
Windows XP	*All Versions no longer supported after April 8, 2014*	–	X
Office 365	All versions	X	–
	*All versions no longer supported after October 2023*	–	–
**BROWSERS**	**Automatic updates by default**	**Manual update**	**Need to be set-up to automatically update**
Google	X	–	–
Safari	X	–	–
Microsoft Edge	X	–	–
Firefox	X	–	–
Internet Explorer	X	–	–
Maxathon	X	–	–
Pale Moon	X	–	–
Avast	X	–	–
Vivaldi	X	–	–
Opera	X	–	–
Tor	–	X	–
Konqueror	–	X	–
Chromium	–	X	–
Falkon	–	X	–
Brave	–	–	X
**EMAIL software**	**Automatic updates**	**Manual updates**	**–**
Go Daddy	–	X	–
HostWinds	–	X	–
Front	–	X	–
Gmail	X	–	–
Apple Mail	X	–	–
Proton	X	–	–
Outlook	X	–	–
Yahoo	X	–	–
MDaemon	X	–	–
OfficeSuite	X	–	–
**SMART PHONES**	–	–	–
Android based	Some X	Some X	–
iOS	X (but you require access to wifi and not mere data usage)	–	–
Windows 10 (older Nokia and Microsoft phones)	*No security patches*	–	–

## Australia

On 19 June 2020, the Prime Minister of Australia revealed that a wide range of Australian political and private-sector organisations were under a malicious cyber-attack by a sophisticated state-based actor.^10^ During its investigation of this incident, the Australian Cyber Security Centre (ACSC) identified that prompt patching of internet-facing software, operating systems, and devices would have greatly reduced the risk of compromise.^11^

Despite exploitation of publicly known vulnerabilities in unpatched software being one of the leading cyber threats,^12^ there is a dearth of cybersecurity regulation and case law in Australia that specifically requires vendors and organisations to implement security updates in a timely manner. Software vendors are able to avoid contractual liability for the damages caused by vulnerabilities in their software through broad contractual exclusions and limitations of liability. Tort law has likewise not materially developed to impose liability on vendors for damages suffered by insecure software^13^ other than in limited circumstances in Australia. Australia has an uneven patchwork of data protection laws and sector-specific laws that broadly recommend compliance with voluntary frameworks, industry standards, and guidelines.^14^

### *Privacy Act 1988* (Cth) and related guidance

The Privacy Act 1988 (Cth) (Privacy Act) is the primary legal instrument that regulates how Australian Government agencies and private-sector organisations with annual revenues over $3 million handle personal information.^15^ The Act does not apply to small businesses which are defined as having annual turnovers of $3,000,000 or less.^16^

The Privacy Act contains 13 Australian Privacy Principles (APPs), which govern standards, rights and obligations around how personal information may be collected, used, disclosed, stored, transferred, accessed, and corrected. A breach of an APP constitutes an ‘interference with the privacy of an individual’ and can be the subject of regulatory action and penalties from the Office of the Australian Privacy Commissioner (OAIC).

One of the APPs that may be relevant in the context of governing organisations’ conduct in patching is APP 11, which requires entities that hold personal information to take reasonable steps to protect the information from misuse, interference and loss, as well as unauthorised access, modification or disclosure.^17^ The Privacy Act does not define what constitutes ‘reasonable steps’ to secure personal information. The OAIC has provided detailed guidance on reasonable steps that entities are expected to take under the Privacy Act to protect personal information in its *Guide to securing personal information*. While the guide is not legally binding, the OAIC is empowered to refer to this guide when investigating whether an entity has complied with its obligations under the Privacy Act.^18^

The guide indicates that what constitutes reasonable steps to ensure information security under the Act will depend on the circumstances. In the digital environment, reasonable steps should include taking steps and implementing strategies in relation to ICT security.^19^ In particular, it recommends that organisations regularly review their software security to confirm its continued effectiveness, test the software to detect security vulnerabilities, and ensure that the latest versions of software and applications are in use. Most importantly, organisations must have processes and procedures in place to ensure that patches and security updates to applications and operating systems are installed as they become available.^20^

The OAIC Guide also advises organisations to consider adopting relevant international and Australian standards, handbooks, manuals, and policies on information security. For example, they may consult the ISO/IEC 27000 series of information security management standards and the ISO/IEC 31000 series of risk management standards, parts of which have been adopted by Standards Australia.^21^ ISO 27001:2015 Annex 12.6.1 Management of Technical Vulnerabilities requires that “information about vulnerabilities of information systems shall be obtained in a timely fashion” and “appropriate measures taken to address the associated risk”.^22^ Arguably, the ‘appropriate measures’ include patching known vulnerabilities.^23^ It should be noted that complying with a voluntary standard does not of itself constitute ‘taking reasonable steps’ to protect personal information for the purpose of the Privacy Act.^24^

The OAIC indicates that organisations must also ensure that security patches are applied as soon as they are released by the vendors. Applications must also be upgraded to newer versions that have more recent security features in a timely fashion, for the reason that older versions are generally known to have unpatched vulnerabilities. The next part will discuss two notable investigations initiated by the OAIC that concern poor patch management practices.

#### Cupid Media Pty Ltd: own motion investigation report

On 21 January 2013, Cupid Media Pty Ltd (Cupid) identified a rogue file on one of its webservers. Cupid then conducted internal investigations and identified that on 18 January 2013, a vulnerability within the application server platform that Cupid used (ColdFusion) was exploited by hackers, which gave them access to Cupid’s webservers. The hackers then uploaded a shell ‘ColdFusion Markup’ (CFM) file that allowed them to run SQL queries against Cupid’s databases and gain unauthorised access to accounts and personal information of approximately 254,000 Australian users.^25^

A patch for the ColdFusion vulnerability was made available on 16 January 2013. However, Cupid did not receive an alert from the developer that the patch was released. Through its business-as-usual internal patch management processes, Cupid’s IT team identified that the patch was available on 21 January 2013 and applied the patch on the same date, thus prevented further data from being compromised in the breach. Cupid also notified affected individuals and ensured they reset their passwords.

The OAIC considered that the information and patch management steps taken by Cupid were reasonable security steps for the purposes of APP 11 in the circumstances. It further emphasised that effective and prompt application of patches can assist organisations to better handle system vulnerabilities and prevent data breaches. Had Cupid received a notification that the patch was available but not applied the patch immediately, the OAIC would have considered that Cupid had failed to take reasonable steps to secure personal information.

However, the passwords of Cupid’s user accounts were stored in plain text. The OAIC therefore found Cupid’s password encryption strategies to be inadequate and a failure to take reasonable steps for the purpose of APP 11.

Based on the information from Cupid about its review and remediation action, the OAIC decided to close the investigation. Cupid did not face any penalties or regulatory action.

Whilst the OAIC did not comment on the developer’s liability (if any) in failing to notify Cupid of the patch availability, it could be argued that had the developer sent Cupid an alert that the patch was released as they ordinarily had been doing, Cupid would have been able to remedy the flaw on the day it identified the data breach.

#### AAPT and Melbourne IT: own motion investigation report

On 6 August 2012, the OAIC opened an investigation into AAPT Ltd (AAPT) and Melbourne IT Ltd (Melbourne IT) following media reports that a server holding AAPT customer information was compromised by the hacker group Anonymous.^26^ By exploiting a vulnerability in the ColdFusion application installed on the server, Anonymous gained unauthorised access to websites and databases on the compromised server that included personal information about AAPT customers, which Anonymous then published on the internet. The server was managed by WebCentral Pty Ltd, a webhosting business unit of Melbourne IT.

At the time of the incident, security patches were up to date on the ColdFusion application. However, AAPT was using a seven-year-old version of the application that was widely known to have vulnerabilities. Several newer versions of ColdFusion were available, the most recent of which had security features that may have prevented the attack. It was also unclear whether AAPT was aware of what ColdFusion applications were installed, and who was responsible for addressing data security issues on the ColdFusion applications.

Given these inadequate safeguards, the OAIC concluded that AAPT had contravened APP 11 for its failure to take reasonable steps to appropriately manage and secure personal information it held from misuse and loss and from unauthorised access, modification, or disclosure. The OAIC recommended that AAPT conduct regular reviews and vulnerability testing of all IT applications, undertake further training of IT staff, and carry out an audit to determine responsibility for identifying and addressing data security issues (such as vulnerabilities associated with old versions of IT applications).^27^ Similar to Cupid, AAPT did not face any penalties or further regulatory action.

As can be seen from the above Cupid Media and Melbourne IT investigation reports the OAIC did not issue a fine on the parties at fault despite infringements of the Privacy Act. However, it should be noted that these investigations occurred in 2012 and 2013, a year before the Privacy Act was amended to give the OAIC enhanced powers to seek civil penalties in cases of serious or repeated breaches of privacy.^28^ There have not been any recent cases concerning businesses’ poor patch management resulting in regulatory action by the OAIC. Nine years on, the Privacy Act is again going through changes as the Australian Government announced major reforms in the wake of increased scrutiny of social media platforms, including additional powers for the OAIC to issue infringement notices and tougher penalties to bring Australia more in line with the General Data Protection Regulation (GDPR).^29^

### Sector-specific: *Corporations Act 2001 *(Cth)

To operate a financial services business in Australia, an organisation or individual must hold an Australian Financial Services (AFS) Licence. The conduct of AFS Licensees is governed by the *Corporations Act 2001 *(Cth) (Corporations Act) and regulated by the Australian Securities and Investments Commission (ASIC). AFS Licensees must comply with the general obligations imposed on them under section 912A(1) of the Corporations Act. These obligations include an obligation to have adequate resources (including financial, technological, and human resources) to provide the financial services covered by the Licence.^30^

ASIC’s Regulatory Guide 104: *AFS licensing: Meeting the general obligations* describes the obligation to have adequate technological resources as encompassing an obligation to regularly review IT systems. When reviewing their IT systems, AFS Licensees must consider:

- their IT system security;
- the currency of their hardware and software;
- the quality and relevance of the applications they use;
- their disaster recovery system and business resumption capacity;
- the number of users;
- the ongoing viability of software and other service providers;
- the response times and down times of their IT systems;
- their use of legacy IT systems; and
- complaints about their IT systems.^31^

The Regulatory Guide does not comment on whether the above obligation extends to a specific requirement that AFS Licensees must remedy security vulnerabilities in a timely fashion.

Although cybersecurity has been an increasing focus for ASIC in recent years,^32^ it only started taking a proactive approach in prosecuting financial services firms for inadequate cybersecurity practices last year. On 21 August 2020, ASIC commenced Federal Court proceedings against RI Advice Group Pty Ltd (RI) alleging AI’s (artificial intelligence) failure to have and implement adequate cybersecurity systems contravened section 912A of the Corporations Act.

#### ASIC v RI advice: AFS licensee sued for inadequate cybersecurity systems

RI is a financial planning and advisory firm and an AFS Licensee. ASIC’s action was the result of a series of alleged cyber breach incidents that occurred to certain authorised representatives (ARs) of RI between 2016 and 2020. These incidents included:^33^

- A ransomware attack that encrypted RI’s files and made them inaccessible.
- A malicious actor obtained and retained unauthorised remote access to RI’s network, affecting 226 client groups.
- A malicious actor used a brute force attack on an RI employee’s account to access RI’s server. The actor spent more than 155 h logged on to the server, using client’s identification documents to redirect a client’s mail with Australia Post and opened multiple bank accounts without the client’s consent.
- An unknown party obtained unauthorised access to the laptop of another RI employee by installing a Trojan virus. This party used the employee account to email the employee’s bookkeeper to request a fund transfer to a Turkish bank account, which was not made.
- An RI’s staff member’s mailbox account was compromised.
- A particular RI’s email account was attacked twice, with over ten thousand emails accessed without authorisation.

Of particular importance is a report about the incident at Frontier, which found major deficiencies in Frontier’s cybersecurity systems, including that:

- 90 Per cent of desktops operated without up-to-date antivirus software;
- there were no scheduled scans for antivirus software;
- there was no filtering or quarantining of emails;
- there were no offsite backups;
- the domain Administrator account was still default and the password was known by external parties;
- passwords and other security details were found in plain text files on the server desktop; and
- the remote desktop computer was accessible on default port.^34^

ASIC alleges that RI contravened sections 912A(1)(a), (b), (c), (d), and (h) of the Corporations Act due to its failure to have and to have implemented (including by its annual reports, policies, plans, procedures, strategies, standards, guidelines, frameworks, systems, resources and controls which were reasonably appropriate to adequately manage risk in respect of cybersecurity and cyber resilience (Minimum Cybersecurity Requirements).^35^ In particular, ASIC alleges that RI should have had in place a detailed plan and steps for identifying, assessing, and applying patches in a routine and formalised manner in order to meet the Minimum Cybersecurity Requirement in respect of Vulnerability Management.^36^

Additionally, in considering what RI should have done, ASIC made specific reference to the Strategies to Mitigate Cyber Security Incidents, a voluntary guideline issued by the Australian Cyber Security Centre (ACSC).^37^ The ACSC noted that it is essential for organisations to patch vulnerabilities as quickly as possible in a timeframe that is commensurate to the level of risk posed to system and applications if no patch is applied. For example, systems with vulnerabilities that are publicly known (“extreme risk”) must be patched within 48 h. Organisations should also use the latest versions of applications that are vendor-supported with patches for security vulnerabilities.^38^ Although ACSC’s voluntary guidelines are not legally binding, ASIC clearly considered them highly persuasive in prosecuting AFS Licensees for poor cybersecurity practices.

ASIC is seeking pecuniary penalties of $11 million or 10% of RI’s parent company, IOOF Group’s, annual turnover (whichever is larger). ASIC also wants a compliance order requiring RI to implement appropriate cybersecurity measures within 3 months of ruling, and to engage an independent expert to confirm RI’s compliance within 5 months.^39^

This is the first known regulatory action brought by ASIC against an AFS Licensee for allegedly inadequate cybersecurity practices. Although this proceeding is still on foot and no judgments have been passed, it emphasises ASIC’s emerging approach to more aggressively hold AFS Licensees accountable for poor cybersecurity risk management systems. Further, a judgment in ASIC’s favour may ultimately expand the scope of general obligations under section 912A to specifically include an obligation on financial services firms to identify, assess, and apply security patches in a routine and formalised manner.

### Sector-specific—*Code of Practice: Securing the Internet of Things for Consumers*

On 3 September 2020, the Australian Government released the *Code of Practice: Securing the Internet of Things for Consumers *(the Code). The Code, which forms part of Australia’s 2020 Cyber Security Strategy, is intended to be a voluntary set of measures the Australian Government recommends for manufacturers, service provides, mobile application developers, and vendors as the minimum standards for Internet of Things (IoT) devices.^40^ It is anticipated that the Code will help facilitate industry best practice with regards to security safeguards and increase consumer confidence in IoT technology.^41^ The Code comprises 13 principles, with the top three considered to be of the utmost importance: no duplicated default or weak passwords, vulnerability disclosure, and security updates.^42^ Of particular relevance to this paper is the third principle—*Keep software securely *updated, which requires that:

- software (including firmware, third party and open source software) on IoT devices, as well as associated webservices, should be securely updateable;
- updates must be installed in a timely fashion;
- updates should not impact the device’s functionality, or change user-configured preferences, security or privacy setting without user’s prior consent;
- consumers must receive clear notification and explanation for the need for each update;
- updates should be easy to implement and applied automatically by default;
- updates should be distributed from a trusted source and via secure IT infrastructure to prevent compromise.^43^

For devices that cannot be updated, the Code requires device manufacturers, service providers, and mobile application developers to inform users that the device is no longer fit for purpose. These parties should also provide an end-of-life policy that explicitly stipulates the minimum length of time for which a device will receive software updates, the reasons for this timeframe, and the vendor’s commitment and method to warn consumers when the product will no longer receive updates.^44^

While the Code represents an important first step towards strengthening IoT devices’ security, some doubt that a voluntary code is sufficient incentive for developers, manufacturers, service providers, and vendors to adopt industry best practice. In addition, the Code may soon become obsolete as the Australian Government looks to keep up with recent regulatory developments in the United Kingdom and United States of America. Last year, the United Kingdom Government proposed to mandate security in consumer smart products only two years after their introduction of a comparable voluntary *Code of Practice for Consumer IoT Security,*^45^ while Californian legislators passed America’s first IoT security law, SB 327, which commenced on 1 January 2020.^46^

### Sector-specific: *Australian Competition and Consumer Act 2010* (Cth)

For consumer protection matters generally, the *Australian Competition and Consumer Act 2010* (Cth) (ACCA) (formerly the *Trade Practices Act 1974*) governs the relationships between consumers, suppliers, wholesalers, and retailers. The ACCA is administered by the Australian Competition and Consumer Commission (ACCC). The general prohibitions in schedule 2 of the ACCA (commonly referred to as Australian Consumer Law or ACL) may be relevant to allow the ACCC or consumers to bring proceedings against a company that fails to timely apply security updates or use the latest versions of applications.^47^ These prohibitions include: *misleading or unconscionable conduct in trade or commerce*^48^ and *unfair contract terms*.^49^ The ACL also creates a set of guarantees for consumers who acquire goods and services from Australian suppliers, importers or manufacturers,^50^ including a *right to repair of goods*.^51^ The ACL consumer protection regimes are mandatory and cannot be excluded, restricted or modified by contract.^52^

In contrast to case law that fell short of delineating whether software which is not attached to a tangible moveable component (e.g., a computer) could be classified as a good,^53^ section 2(1) of the ACL expressly states that “computer software” on its own constitutes a ‘good’ for the purpose of the ACL. It is also interesting to note that the ACL requires only that there be a ‘supply’ of goods, including re-supply by way of ‘sale, exchange, lease, hire or hire-purchase’.^54^ This means it covers software that is licensed and not sold, such as digitally downloadable computer games,^55^ operating systems, and web-based software.^56^

#### Misleading or unconscionable conduct

So far, the authors are not aware of any actions in Australia brought by either consumers or the ACCC alleging misleading or unconscionable conduct of organisations that publicly claim to keep their systems and applications securely patched and are then proven not to be. One possible reason for the lack of claims is a lack of consumer knowledge regarding whether the ACL provisions apply to their cases. Recent regulatory action instead points to a greater application of the Privacy Act and the Corporations Act, as discussed in parts A and B. Another possible reason is that consumers may choose to complain directly to the manufacturers or retailers and privately settle for a refund or repair instead of going through expensive and lengthy proceedings.

Despite the absence of private or public claims under these provisions against companies for poor patch management, some academics still consider the ACL a viable instrument in providing consumer redress against businesses’ lack of correction of known vulnerabilities in their products.^57^ The Federal Trade Commission, the US equivalent of the ACCC, has succeeded in a proceeding against a company for substandard cybersecurity measures, which included the use of an out-of-date operating system that had not received a security update in over three years.^58^

#### Unfair contract terms

Another general protection that may be available to consumers and the ACCC relates to unfair terms in standard form consumer contracts.^59^ Although the ACL does not define a ‘standard form contract’, it is typically a contract that has been unilaterally prepared by one party with all the bargaining power and is not subject to negotiation between the parties—that is, it is offered on a ‘take it or leave it’ basis.^60^ In that sense, license agreements between manufacturers and end users are likely standard form contracts and thus subject to this provision. Their terms will be considered ‘unfair’ if they:

- would cause a significant imbalance in the parties’ rights and obligations;
- are not reasonably necessary to protect the legitimate interests of the party who would be advantaged by the term; and
- would cause detriment (whether financial or otherwise) to a party if it were to be applied or relied on.^61^

Section 25 of the ACL provides a non-exhaustive list of terms that may be unfair. An example of a potentially unfair term is one that ‘limits, or has the effect of limiting, one party’s right to sue another’.^62^ In the context of security updates, such a term may attempt to expressly prohibit end users from suing the manufacturers for damages suffered caused by the use of older products no longer supported with security updates. If a court finds the term unfair, it is void.^63^

#### Consumer guarantees: right to repair

With regards to consumer guarantees, of particular relevance is the guarantee as to repairs. Manufacturers of ‘goods’, which expressly include ‘computer software’ under the ACL,^64^ must have spare parts and repair facilities ‘reasonably available’ for a ‘reasonable’ period.^65^ Unfortunately, the ACL does not explain what ‘reasonably available’ means and how long the “reasonable period” is supposed to be in practice, nor does it provide any criteria that may be used to determine the duration of such a period.^66^

Concerns have been raised in recent years that Australian consumers face unnecessary barriers to accessing repair services, including barriers caused by manufacturers making products rapidly become obsolete and require a replacement or upgrade, or refusing to service products that were previously serviced by non-authorised repairers.^67^ One well-known example of such conduct occurred when the Federal Court of Australia found that Apple misled customers in relation to their entitlement to repairs under the ACL.^68^ In 2018, the ACCC commenced proceedings against Apple following investigation into customers’ complaints about ‘error 53’. This error disabled some iPhones and iPads after customers downloaded an update to Apple’s iOS operating system. Between February 2015 and February 2016, Apple informed customers that they were no longer entitled to remedies (repair, refund or replacement) for ‘error 53’ if their device had been repaired by an independent repairer. The Federal Court declared that the fault was covered by consumer guarantees and that ‘the mere fact that an iPhone or iPad had been repaired by someone other than Apple did not, and could not, result in the consumer guarantees ceasing to apply’. Consequently, Apple paid $9 million dollars in penalties and compensated 5000 customers.^69^

There are limits to how software users can use this consumer guarantee to demand timely installation of patches post-sale. First, manufacturers can ‘opt out’ by advising the consumer at the time of supply that repair facilities and spare parts will not be available after a specified time.^70^ In the current context, this means software manufactures are not permitted to stop providing security updates to older versions of their products (sometimes as a way to force their customers to upgrade to new versions with the latest updates), 71unless they inform customers at the time of purchase about the specified time for end of support.

Another problem with this repair guarantee is that it is only available to a ‘consumer’. Section 3 of the ACL currently provides that a person or a business is taken to be a ‘consumer’ if they have purchased goods or services for less than $40,000 (this threshold will increase to $100,000 from 1 July 2021), or more than $40,000 for goods or services that are of a kind ordinarily acquired for domestic, household, or personal use or consumption. 72The main takeaway from this definition is that the person or business must have paid a consideration for the goods or services. Since much software and many Internet services are ‘free’ (where the vendors do not generate revenue from licensing fees paid by users, but by monetizing user data, for example), the users of such software and services cannot be considered a ‘consumer’. In that case, it is unlikely that they will be able to demand prompt patches or ongoing updates for free unsupported software and services under the ACL repair guarantee. Additionally, users will also be ineligible for this guarantee if they pay more than $40,000 for software or services that were not used in their private capacity, but rather for commercial or business activities.

### Conclusion

Overall, the legal obligation to patch security vulnerabilities is practically non-existent in the Australian model of cybersecurity regulation. While a number of legislative provisions could, in theory, enable a regulator or consumer to take action against organisations for failure to patch known vulnerabilities in their systems and applications, few have actually been utilised and tested in courts. In the absence of judicial guidance, the regulators are possibly reluctant to go to courts, and instead rely on voluntary guidelines and frameworks. While these guidelines are highly persuasive, they are not legally binding and are unlikely to be adequate incentive for compliance. Unless the regulators take a more aggressive approach to hold organisations accountable in courts, the vicious cycle of limited judicial guidance and legal uncertainty continues.

ASIC’s proceeding against RI signals, however, a new regulatory approach to cybersecurity by regulators in other sectors, considering that this proceeding takes place against the broader backdrop of a heightened government focus on cyber security. The Commonwealth recently announced Australia’s Cyber Security Strategy 2020, which focuses on reforming Australia’s laws to ensure that the Commonwealth has the appropriate safeguards and powers to uplift the security and resilience of critical infrastructure.^73^ The Cyber Security Strategy also specifically states that businesses must “take responsibility for enhancing their own cybersecurity, just as they are responsible for the safety and quality of their products”.^74^ It is also expected that some of the current voluntary guidelines issued by the Australian Cyber Security Centre—including recommendations for businesses to patch security vulnerabilities as quickly as possible^75^—will be mandated to enhance positive cyber security obligations.^76^ At present, however, this has not happened.

##  European Union (EU)

The European Union has implemented a wide spectrum of legal instruments, proposals, and initiatives that seek to address various issues in cybersecurity, ranging from data protection regulations to directives concerning network and information security. Not unlike Australia, the EU’s cybersecurity legal framework has also been characterised by critics as patchwork, lacking in consistency and strategic cooperation among Member States.^77^ As far as a legal obligation to patch is concerned, there are no objective or minimum standards or mechanisms clearly defined or mandated within EU legislation to oblige vendors and businesses to promptly patch vulnerabilities. In the next section, we identify a number of legal instruments in the EU, either already implemented or proposed, that to some extent make reference to a legal obligation to patch.

### EU Cybersecurity Act of 2019

The EU has recently taken one step further towards harmonisation in the field of cybersecurity law at international level. Following the EU’s Communication on Strengthening Europe’s Cyber Resilience System and Fostering a Competitive and Innovative Cybersecurity Industry in 2016,^78^ the Regulation (EU) 2019/881 of the European Parliament and of the Council of 17 April 2019 on ENISA (European Union Agency for Cybersecurity) and on information and communication technology cybersecurity certification and repealing Regulation (EU) No 526/2013 (Cybersecurity Act), came into force on 27 June 2019.^79^ The new Regulation aims to achieve a higher level of cyber resilience and strategic cooperation within the EU and improve Member States’ capabilities to prevent, detect, and respond to cyber threats, including cross-border incidents.^80^

The Regulation has two main objectives: (1) to further mandate and strengthen the role of the European Union Agency for Network and Information Security (ENISA, the EU Agency for cybersecurity);^81^ and (2) to establish an EU-wide certification framework for ICT digital products, services and processes.^82^ The purposes of the certification framework are to harmonise cybersecurity practices within the EU,^83^ and protect the availability, authenticity, integrity, or confidentiality of stored, transmitted, or processed data and the functions or services offered by ICT products, services, and processes throughout their life cycle.^84^ It consists of cybersecurity certification schemes that introduced common requirements and evaluation criteria for digital products that are recognised within the EU. Once the schemes are fully developed, manufacturers, suppliers, and vendors will have the opportunity to certify that their ICT products and services meet EU uniform cybersecurity standards.

As far as a patching obligation is concerned, the Regulation states that the certification schemes shall be designed to ensure that ICT products, services, and processes are provided with up-to-date software and hardware that do not contain publicly known vulnerabilities, and are provided with mechanism for secure updates.^85^ Further, manufacturers and providers of these products should provide any necessary updates and should recall, withdraw or recycle products that do not meet cybersecurity standards, while importers and distributors should make sure that the products they place on the EU market comply with applicable security requirements and do not present a risk to EU consumers.^86^ In addition, manufacturers or providers of digital products, services, and processes should provide end users with information about their cybersecurity support policy, namely for how long end users can expect to receive cybersecurity updates or patches.^87^ Should the manufacturers or providers fail to comply with their obligations arising from the Regulation, they will face warnings from ENISA,^88^ complaints lodged with Member States’ national cybersecurity certification authorities, withdrawal of their security certification, and financial penalties.^89^ These consequences of non-compliance entered into force on 28 June 2021.^90^

While the new Cybersecurity Act is a welcome legislative development, the cybersecurity certification scheme it established is voluntary.^91^ Unless Member States adopt the scheme into national laws, or the EU mandates it and applies strict enforcement of the obligations arising out of the scheme and the Cybersecurity Act, it is unlikely there will be any consequences for firms who fail to patch known vulnerabilities in their uncertified products.^92^ The good news is, the European Commission will regularly assess the effectiveness of the adopted schemes and whether mandatory certification is required for certain types of products and services.^93^

### General Data Protection Regulation (GDPR)

The protection of personal data in the EU is governed by Regulation (EU) 2016/679 of the European Parliament and of the Council of 27 April 2016 on the protection of natural persons with regard to the processing of personal data and on the free movement of such data (GDPR), which came into effect on 25 May 2018. The GDPR contains new data protection requirements that replaced national data protection rules under the 1995 Data Protection Directive (Directive 95/46/EC) and harmonise data protection laws across the EU.

Similar to the Australian Privacy Act, the GDPR requires businesses to implement appropriate security measures to prevent the personal data they hold from being deliberately or accidentally compromised. Article 32 of the GDPR states that organisations “shall implement appropriate technical and organisational measures to ensure a level of security appropriate to the risk”. Recital 78 repeats the requirement for appropriate technical and organisational measures. The GDPR does not specifically define “appropriate technical measures” or give examples, but trends in enforcement action by EU data protection authorities clearly indicate that regular software updates and using the latest version are part of the required measures.^94^ Failure to implement these measures therefore constitutes violation of GDPR article 32.

Although the United Kingdom (UK) is no longer part of the EU, the UK’s Information Commissioner’s Office (ICO) has investigated (on behalf of EU’s data protection authorities) and issued several GDPR fines to organisations for poor vulnerability handling practices that contravened article 32:

- £400,000 fine issued to Carphone Warehouse for insufficient software patching;^95^
- £500,000 fine issued to DSG Retail in January 2020 for inadequate software patching, outdated Point-of-Sale software, and infrequent vulnerability scanning;^96^
- £500,000 fine issued to Cathay Pacific in March 2020 for unpatched internet-facing servers, use of operating systems that were no longer supported by the developer, and inadequate anti-virus protection^97^ and
- £20m fine issued to British Airways (BA) in October 2020 for a cyber-attack that occurred in 2018 due to BA’s failure to update JavaScript, failure to identify a well-known and preventable security vulnerability, and lack of effective monitoring of potential vulnerabilities.^98^

Other than the UK ICO, the Norwegian DPA also reprimanded Telenor in February 2021 for unpatched vulnerabilities in its voicemail function that have been known for many years.^99^

### Directive on security of network and information systems (NIS directive)

The NIS Directive, which came into force in August 2016, is the first piece of EU-wide legislation on cybersecurity.^100^ Member States had transposed it into their respective national strategy on the security of network and information systems.^101^ The NIS Directive regulates operators of essential services, which are services that are essential for the maintenance of critical societal and/or economic activities.^102^ Such operators as well as digital service providers are obliged to take “appropriate proportionate technical and organisational measures” to manage risks and provide a level of security appropriate to the risk posed.^103^

The directive is silent as to what precisely constitutes “appropriate and proportionate technical and organisational measures”. However, in accordance with article 19(2) of the directive, ENISA has issued a set of non-binding guidelines to assist Member States in providing a common approach regarding the required technical and organisational measures.^104^ The guidelines recommend that operators establish and maintain appropriate procedures for testing key network and information systems that support their services and ensure software security, which explicitly include checking for and installing the latest patches.^105^ Commentators also agree that patching is one of the practical measures that organisations should apply to effectively respond to cyber risks that unpatched vulnerabilities pose to their systems and to ensure compliance with the NIS directive.^106^ Some went further to argue that this provision should be interpreted as creating the presumption of liability for negligence against digital service providers/operators in the case where a user suffers damage from a cyber-attack through a provider/operator’s information system.^107^

An evaluation by the European Commission on the efficiency of the NIS Directive identified a number of limitations: businesses in the EU have a low level of cyber resilience; Member States and sectors’ resilience is inconsistent; and there is a low level of joint situational awareness and lack of joint crisis response.^108^ To address these deficiencies of the current NIS Directive, a revised NIS Directive (NIS 2) was proposed on 16 December 2020, which will build on and repeal the current NIS Directive.^109^ In terms of patching requirement, the new legislative proposal is intended to impose a risk management approach that introduces a minimum list of basic security measures that companies must apply,^110^ which include measures relating to “vulnerability handling and disclosure”.^111^ Further, the proposed directive creates an European registry of known vulnerabilities and related patches maintained by ENISA where organisations, whether regulated by the Directive or not, may choose to disclose vulnerabilities and provide the vulnerability information that allows users to take appropriate mitigating measures.^112^

###  Dutch Consumers’ Association (*Consumentenbond*) v. Samsung

In November 2016 (before the GDPR), the Dutch Consumers’ Association (DCA) sued Samsung in the District Court of the Hague, Netherlands, demanding that Samsung provide timely Android updates for their smartphones for four years after launch, or at least two years from the date of purchase. The DCA also called for Samsung to increased transparency and communication in its software update schedule.^113^ The Court ultimately ruled in Samsung’s favour that it does not have to provide more than two years of updates.

It should be noted that Android is an open-source operating system developed and operated on Google. Google regularly checks for vulnerabilities in Android and creates a patch if a bug or security vulnerability is found, and Google makes updates available to manufacturers of smartphones such as Samsung. These manufacturers can test and modify the updates if necessary, then distribute to their users for download and installation.^114^ Often, not all devices will receive the updates, which could be because the specifications of the device are insufficient to support the patch or because the device’s performance would significantly deteriorate due to the update.^115^ However, there are some companies who simply do not implement the patches in question, and when they do release a patch, not all of these are automated, requiring a human to manually implement the update.

#### The DCA’s allegations

The DCA’s allegations in this case were that Samsung breached its duty of care as expected of it in society or were in breach of its obligations arising from:^116^

- Article 7:17 of the Dutch Civil Code^117^ and/or Article 13 of the Dutch Personal Data Protection Act by failing to provide its smartphones with updates in a timely manner, at least for a period of four years from the device’s introduction to the market or at least within a month of becoming aware of a vulnerability and the availability of Google’s patch for that vulnerability. The DCA argued that many Samsung devices did not run on the latest version of Android, and that Samsung only makes monthly updates available for a select number of devices and its other devices receive updates with a long delay or no updates at all.^118^
- Article 6: 193d and/or Article 6: 193 of the Dutch Civil Code by failing to advise consumers before their purchase of a Samsung smartphone, at least not in a clear and unambiguous manner, about:
  i. The version of the operating system and/or whether this is the most recent version;
  ii. Whether the device receives software updates and/or upgrades;
  iii. How the device’s expected software support period will affect the consumer;
  iv. The period within which the consumer can expect to receive such updates or upgrades.

The DCA requested that the Court ordered Samsung to:^119^

- provide all Samsung smartphones in the Netherlands with updates that fix software vulnerabilities for a period of four years after a device enters the market and/or two years after a device’s purchase,
- fix security vulnerabilities in the Android operating system within one month after Google makes the update available, and
- inform consumers in a clear and unambiguous manner before each device’s sale about Samsung’s update and upgrade policy.

#### The court’s decision

In a decision handed down on 30 May 2018, the District Court of The Hague noted that to succeed in this claim, the DCA must prove that (i) that there is a real security risk for users of Samsung smartphones, and (ii) that Samsung does in fact not do enough to alleviate those risks because it does not in all cases carries out timely upgrades and updates.^120^

With regard to the first element, the Court found that the DCA did not sufficiently prove that any vulnerability identified by Google would automatically expose users of Samsung smartphones to real security risks. It could not be assumed that such vulnerability would automatically lead to actual danger to these consumers.^121^

The second question for the Court was whether Samsung, in view of the security risks that may be associated with vulnerabilities in Android that Google had identified, was not doing enough to counteract these risks with timely implementation of upgrades and updates. It was not disputed that Samsung should take adequate and effective action against the security risks. What would be sufficient, adequate, and effective would depend on the nature and severity of the actual/real safety risks in the circumstances.^122^

The Court noted that Samsung relied on Google to provide patches for the Android core to develop updates; however, Samsung must adjust and test the Google patches for each released model before the update could be downloaded and installed by users. It followed that the actual turnaround time for an update was not always the same but depended on, among other factors, the number of patches to be processed, the technical complexity of the update, and the capability of each model to receive the update. Samsung’s view was that a device with better specifications (often a new model) would have a greater chance of successful update installation.^123^ Samsung also argued, which the Court accepted, that it could not update all of its smartphones at the same time, but must instead set priorities based on actual threat level and technical as well as economic considerations. Samsung further argued, which was not contradicted, that it was important that there were no official professional standards for providing updates.^124^

In light of the above considerations, the Court ruled that the DCA did not present sufficient evidence to prove that Samsung was doing too little to address the security risks of vulnerabilities in Android, nor was it successfully established by the DCA that Samsung exposed its consumers to a real risk of possible harm by malicious parties by failing to patch all of its devices. It was also not possible to order Samsung to update all smartphones for four years without knowing what flaw may be discovered in the future and whether the patch could effectively fix the flaw.^125^

Regarding the DCA’s demand for increased transparency from Samsung, the Court dismissed the DCA’s accusation that Samsung committed unfair commercial practice, more specifically a misleading omission, for failing to inform consumers on its website about the dangers of unsecure smartphones.^126^ The Court considered that Samsung had sufficiently provided information about its update and upgrades policy on its Dutch website following the DCA’s complaint. Specifically, the Dutch website of Samsung displayed a ‘banner’ at the top of the home screen that read: “Samsung provides at least two years of software support for smartphones. Click here for more information.”^127^

#### Implications

This case occurred before the GDPR came into force and was brought and decided on the basis of obligations arising out of the Dutch Civil Code and the Dutch Personal Data Protection Act. If similar claims are brought now, article 32 of the GDPR will likely apply instead.

This case has demonstrated that in claims against technological companies for their failure to patch, generic arguments about future security risks that may or may not materialise are not enough. For these claims to succeed, focus should be placed on collecting concrete evidence about: (i) the security risks and breaches that have occurred and are occurring; (ii) whether, and if so, how timely manufacturers and vendors address these risks through patching the security vulnerabilities; and (iii) the effectiveness of the patches in alleviating the risks, or in other words, how the lack of timely patching has led to the risks being realised to the detriment of the manufacturers and their customers. Doing so will help proving the elements that the Court had identified above: (i) that there is a real security risk for users, and (ii) that the vendor does not do enough to lessen those risks because it does not in all cases carry out timely upgrades and updates.

##  Reform proposals

As has been observed in the above discussion of the legal landscape in Australia and the European Union, there have been important developments in the legislative framework governing the conduct of software vendors and companies in relation to information security. While these developments are heading in the right direction towards strengthening cyber resilience generally, they still fall short of imposing a legal obligation on vendors and companies to correct known vulnerabilities promptly; to move to automatic security updates by default; and to require companies to support their products. Further, there is no consensus as to how such obligations should be mandated and enforced. There are a number of ways to reform the Australian framework and move towards incentivising timely security patches and automatic updates by default. These include: expanding GDPR-like provisions to include security obligations; contractual remedies; creating a tortious duty to patch; expedited pathways for class action suits; adding a protocol on patching into the EU Cybercrime Convention and/or standards such as ISO Standards.

The irony in Australia is that changes to the European GDPR and Cybercrime Convention may be a good way forward, given their global reach and impact. However, relying on the European Union to lead in privacy and security is a limited option, and does not send a strong message to Australian companies, nor does it adequately protect Australian consumers and businesses.

The Samsung decision provides many important possible lessons. Samsung provides mere notices of its poor security update policy (or lack thereof) on their website which was the sole security requirement for them in 2016, and still the only requirement for them in Australia to date. They only support their product for two years which, from a business perspective, makes sense because they want you to buy a new phone. They do issue security updates, but how often they do so depends on the phone that you own, and where you live. Some products receive a monthly security update, others quarterly, and others bi-annually. The claim is that it isn’t possible to do this on a monthly basis globally. Why can iPhone do this and Samsung cannot? One theory is that they really do not have an economic incentive to do so. Unless the law compels them to take responsibility, they will not do so. Samsung is not alone, and is, in fact, by no means the worst of smart phones using Android. There are thousands of companies just like this globally who act minimally to secure their customers. How do we break the cycle of insecure software, secure patching and move to where security and privacy by design prevail? No one has the answer to this at present but a move towards tightening up security patching requirements is a start.

Any security patching framework requires several essential elements:

- A mandatory unambiguous specified time for product support (Eg. 5 years) excluding technologies specific to critical infrastructure (much higher requirements and outside the scope of this paper).
- Security updates need to adhere to standard of a minimum of monthly updates (if a company cannot adhere to this, then they are likely producing too many products too quickly with insufficient infrastructure).
- Updates need to be automatic by default (manual can be enacted through settings).
- Security updates need to be timely as defined in a manner appropriate to the risk, and feasibility (this will require very careful consideration).
- Compliance mechanisms can be soft or hard. The EU has been progressively moving from the carrot to the stick, as soft mechanisms (self-regulation) have not produced desired changes in security and privacy posture.
- Australia should consider initiating a task-force in the region to develop a regional approach to security patching.

In December 2021 companies scrambled to deal with the very significant Apache log4j security vulnerability—a vulnerability that affected millions of systems globally as it affected Java packages, with Google estimating that 8% of the cyber ecosystem was potentially affected by this vulnerability^128^. The professional community scrambled to release a security patch(es) for the vulnerability, but even more challenging, was—and continues to be—the complexity involved in identifying and patching the problem. Google estimates it will take years to fully patch given how comprehensively it is embedded into the ecosystem. The vulnerability is also open source, meaning that no one company is responsible for the software. This leads us to two problems with legal requirement to timely patching: 1. What is timely? 2. How can you impose a legal requirement for open source development where, ultimately, there are no easily identifiable companies to sue in contract or tort?

Clearly any timely legal requirement to issue a security patch will need to be fully flexible to take into account the complexities involved along with the context but this is not impossible. Having clarity would greatly assist any company in their overall risk management. Because there often isn’t an entity to sue in contract or tort, this should not be the preferred method. There is a movement to demand cybersecurity performance in contracts with governments and larger organisations. Governments and larger organisations could commence the practice of requiring companies to fully support their products for a certain amount of years and with regular security patches. This would simply be implemented as a contractual clause. This, of course, is not a solution for smaller businesses and consumers who rely on governments and larger organisations with influence to help keep them secure.

As Europe moves towards more formal legal frameworks to security systems, Australia should follow suit starting with requirements to implement timely security patches, and by requiring systems to be automatically updated by default. Or better yet, lead the field as we have done in the past by introducing the e‑Safety Commission, requiring Internet Service Providers to take on cybersecurity obligations on behalf of their customers to reduce devices connected to botnets, and through other ground-breaking initiatives that have attracted positive global attention.

## Footnotes

1 Herskovitz, J and Jeong-Ho, L*., A Growing Army of Hackers Helps Keep Kim Jong Un in Power*, Bloomberg. December 22 2021. https://www.bloomberg.com/news/articles/2021-12-21/north-korean-army-of-cybercriminals-props-up-kim-s-nuclear-program-and-economy.

2 This is one of the most common attack vectors often distributed via phishing. In some attack scenario, an attacker employs social engineering to lure the victim into opening a malicious email attachment that will enable the execution of the ransomware payload or install a piece of malicious software allowing the criminal access to an entity’s systems (this can also be used for payment diversion fraud).

3 On the network level, an attacker could gain admin access to server credentials with remote access. Once within the network, the attacker could exploit administrative tools and vulnerabilities to distribute and infect other devices within the network.

4 These are software packages used to create vulnerabilities within a system or network in order to perform malicious activities. For example, Eternal Blue was used in the 2017 WannaCry ransomware attack that infected over 200,000 systems globally.

5 Targeted adverts are usually displayed to potential victims based on their search history or certain web preferences. As an attack vector, malware advertising displays advert with hidden malware links but mirrored as a normal advert specifically placed by a cybercriminal.

6 This vector allows a malware to infect users’ devices by exploiting simple security flaws. Attackers place the malware often on compromised websites, then the malware automatically downloads and installs itself on the victim’s device once the website is accessed.

7 NVD. (2021). *NATIONAL VULNERABILITY DATABASE*. Retrieved from NVD: https://nvd.nist.gov/ and See NIST. (2021). *NATIONAL VULNERABILITY DATABASE*. Retrieved from NIST : https://nvd.nist.gov/vulnmetrics/cvss.

8 Johnson, P. *Curiosity about lines of code.* ITworld. August 8, 2012. Available: http://www.itworld.com/article/2725085/big-data/curiosity-about-lines-of-code.html.

9 Metz, C*. Google Is 2 Billion Lines of Code—And It’s All in One Place.* September 16, 2015. Wired Magazine. Available: https://www.wired.com/2015/09/google-2-billion-lines-codeand-one-place/.

10 https://www.pm.gov.au/media/statement-malicious-cyber-activity-against-australian-networks.

11 https://www.cyber.gov.au/acsc/view-all-content/advisories/advisory-2020-008-copy-paste-compromises-tactics-techniques-and-procedures-used-target-multiple-australian-networks.

12 https://www.cyber.gov.au/sites/default/files/2020-09/ACSC-Annual-Cyber-Threat-Report-2019-20.pdf page 14.

13 Scott, D. *Tort liability for vendors of insecure software: Has the time finally come?* 2008. Maryland Law Review. 67: pp. 425–484.

14 Scott J. Shackelford, Scott Russell & Jeffrey Haut, 2016, *Bottoms up: A Comparison of Voluntary Cybersecurity Frameworks.*

15 *Privacy Act 1988 *(Cth) ss 6(1), 6C(1).

16 *Privacy Act 1988 *(Cth) s 6D(3).

17 *Privacy Act 1988 *(Cth) APP 11.1.

18 https://www.oaic.gov.au/privacy/guidance-and-advice/guide-to-securing-personal-information/.

19 https://www.oaic.gov.au/privacy/guidance-and-advice/guide-to-securing-personal-information/.

20 https://www.oaic.gov.au/privacy/guidance-and-advice/guide-to-securing-personal-information/.

21 https://www.oaic.gov.au/privacy/guidance-and-advice/guide-to-securing-personal-information/.

22 Standards Australia, SO/IEC 27001:2015 Information technology—Security techniques—Information security management systems—Requirements, 1st ed, Published: 04/29/2015.

23 https://www.isms.online/iso-27001/annex-a-12-operations-security/.

24 https://www.oaic.gov.au/privacy/guidance-and-advice/guide-to-securing-personal-information/.

25 https://www.oaic.gov.au/privacy/privacy-decisions/investigation-reports/cupid-media-pty-ltd-own-motion-investigation-report/.

26 https://www.oaic.gov.au/privacy/privacy-decisions/investigation-reports/aapt-and-melbourne-it-own-motion-investigation-report/.

27 https://www.oaic.gov.au/privacy/privacy-decisions/investigation-reports/aapt-and-melbourne-it-own-motion-investigation-report/.

28 https://www.oaic.gov.au/privacy/the-privacy-act/history-of-the-privacy-act/.

29 https://www.ag.gov.au/integrity/consultations/review-privacy-act-1988.

30 *Corporations Act 2001 *(Cth) s 912(1)(d).

31 ASIC, Regulatory Guide 104: *AFS licensing: Meeting the general obligations, *April 2020, RG 104.96.

32 See for example Greg Medcraft (ASIC Chairman), ‘Building resilience: the challenge of cyber risk’ (Australian Chamber of Commerce and Industry business reception event (Melbourne, Australia), 15 December 2016) <https://download.asic.gov.au/media/4120903/speech-medcraft-acci-dec-2016-1.pdf>; ASIC, Cyber resilience of firms in Australia’s financial markets: 2018–19 (Report 651, December 2019) <https://download.asic.gov.au/media/5416529/rep651-published-18-december-2019.pdf>.

33 Concise Statement.

34 Para 71 of Statement of Claim.

35 Para 13 of Statement of Claim.

36 Para 14 and Schedule A of Statement of Claim.

37 Statement of Claim.

38 https://www.cyber.gov.au/acsc/view-all-content/publications/strategies-mitigate-cyber-security-incidents.

39 Concise Statement para 20; Statement of Claim paras 4 to 6.

40 https://www.homeaffairs.gov.au/reports-and-publications/submissions-and-discussion-papers/code-of-practice; https://www.homeaffairs.gov.au/reports-and-publications/submissions-and-discussion-papers/cyber-security-strategy-2020.

41 *Code of Practice: Securing the Internet of Things for Consumers *page 1.

42 See above.

43 *Code of Practice: Securing the Internet of Things for Consumers *page 3, principle 3.

44 Ibid.

45 https://www.gov.uk/government/publications/proposals-for-regulating-consumer-smart-product-cyber-security-call-for-views/proposals-for-regulating-consumer-smart-product-cyber-security-call-for-views.

46 https://leginfo.legislature.ca.gov/faces/billTextClient.xhtml?bill_id=201720180SB327.

47 Stephen Corones and Juliet Davis, ‘Protecting Consumer Privacy and Data Security: Regulatory Challenges and Potential Future Directions’ (2017) 45 *Federal Law Review* 66, 69; Angela Daly, ‘The introduction of data breach notification legislation in Australia: A comparative view’ (2018) 34(3) *Computer Law & Security Review *477, 481. See for example Igor Kossov, LinkedIn Strikes $1.25M Settlement In Data Breach Action, 18 August 2014, https://www.law360.com/articles/568135/linkedin-strikes-1-25msettlement-in-data-breach-action.

48 ACL s 18.

49 ACL s 23.

50 ACL Part 3–1.

51 ACL s 58.

52 ACL s 64(1); Australian Competition and Consumer Commission v Valve Corporation (No 3) [2016] FCA 196.

53 Toby Constructions Products Pty Ltd v Computa Bar (Sales) Pty Ltd [1983] 2 NSWLR 48, 54; Re Amlink Technologies Pty Ltd and Australian Trade Commission (2005) 86 ALD 370, 377 [42]; Gammasonics. Institute for Medical Research Pty Ltd v Comrad Medical Systems Pty Ltd (2010) 77 NSWLR 479.

54 ACL s 54(1)(a).

55 Australian Competition and Consumer Commission v Valve Corporation (No 3) [2016] FCA 196.

56 ACL s 2(1) (definition of ‘supply’); Goldiwood Pty Ltd t/as Margaret Franklin & Associates v ADL (Aust) Pty Ltd t/as Adviser Logic [2014] QCAT 238.

57 Stephen Corones and Juliet Davis, ‘Protecting Consumer Privacy and Data Security: Regulatory Challenges and Potential Future Directions’ (2017) 45 *Federal Law Review* 66, 91; Andrew Smith, ‘FTC Regulating Cybersecurity Post Wyndham An International Common Law Comparison on the Impact of Regulation of Cybersecurity’.

58 F.T.C. v. Wyndham Worldwide Corp., 799 F.3d 236 (2015) https://static.reuters.com/resources/media/editorial/20180607/FTC%20v%20Wyndham%20Worldwide%20Corp.pdf.

59 ACL s 23.

60 https://consumerlaw.gov.au/sites/consumer/files/2016/05/0553FT_ACL-guides_ContractTerms_web.pdf; ACL s 27(2).

61 ACL s 24(1).

62 ACL s 25(k).

63 ACL s 23(1).

64 ACL s 2 (definition of “goods”). Also see Microsoft agreeing that their software constitutes goods for the purpose of ACL under cl 13 of their License Term at https://www.microsoft.com/en-us/Useterms/Retail/Windows/10/Useterms_Retail_Windows_10_English.htm.

65 ACL s 58(1).

66 Consumer guarantees: A guide for consumers, ACCC, https://www.accc.gov.au/system/files/Consumer%20Guarantees%20A%20guide%20for%20consumers_0.pdf; Productivity Commission, ‘Right to Repair’, Productivity Commission Issues Paper, December 2020, page 9. Available at https://www.pc.gov.au/inquiries/current/repair/issues/repair-issues.docx.

67 Productivity Commission, ‘Right to Repair’, Productivity Commission Issues Paper, December 2020, page 9. Available at https://www.pc.gov.au/inquiries/current/repair/issues/repair-issues.docx.

68 Australian Competition and Consumer Commission v Apple Pty Ltd (No 4) [2018] FCA 953. Available at https://www.judgments.fedcourt.gov.au/judgments/Judgments/fca/single/2018/2018fca0953.

69 Australian Competition and Consumer Commission v Apple Pty Ltd (No 4) [2018] FCA 953. Available at https://www.judgments.fedcourt.gov.au/judgments/Judgments/fca/single/2018/2018fca0953.

70 ACL s 58(2).

71 https://www.microsoft.com/en-us/microsoft-365/office-2010-end-of-support;.

72 Treasury Laws Amendment (Acquisition as Consumer—Financial Thresholds) Regulations 2020.

73 https://www.homeaffairs.gov.au/about-us/our-portfolios/cyber-security/strategy.

74 https://www.homeaffairs.gov.au/cyber-security-subsite/files/cyber-security-strategy-2020.pdf.

75 ACSC, Strategies to Mitigate Cyber Security Incidents, 1 Feb 2017, https://www.cyber.gov.au/acsc/view-all-content/publications/strategies-mitigate-cyber-security-incidents.

76 https://www.nortonrosefulbright.com/en/knowledge/publications/5e591584/the-cybersecurity-standards-set-to-impact-every-australian-business-and-director.

77 Maria Garzia Porcedda, “Patching the Patchwork: appraising the EU regulatory framework on cybersecurity breaches”, Computer Law and Security Review, 34, 2018, pp.1077–1098; Agnes Kasper and Alexander Antonov, “Towards Conceptualizing EU Cybersecurity Law”, Discussion Paper C253 2019, page 18.

78 https://eur-lex.europa.eu/legal-content/EN/TXT/?uri=COM:2016:410:FIN.

79 https://eur-lex.europa.eu/eli/reg/2019/881/oj article 69(1).

80 https://eur-lex.europa.eu/eli/reg/2019/881/oj found in multiple articles including articles 6, 25, and 34.

81 https://eur-lex.europa.eu/eli/reg/2019/881/oj articles 16.

82 https://eur-lex.europa.eu/eli/reg/2019/881/oj at (48).

83 https://eur-lex.europa.eu/eli/reg/2019/881/oj at (95).

84 https://eur-lex.europa.eu/eli/reg/2019/881/oj at (75).

85 https://eur-lex.europa.eu/eli/reg/2019/881/oj article 51(j).

86 https://eur-lex.europa.eu/eli/reg/2019/881/oj para (50).

87 https://eur-lex.europa.eu/eli/reg/2019/881/oj para (93).

88 https://eur-lex.europa.eu/eli/reg/2019/881/oj para (58).

89 https://eur-lex.europa.eu/eli/reg/2019/881/oj article 58(8).

90 https ://eur-lex.europa.eu/eli/reg/2019/881/oj article 69(2).

91 https://ec.europa.eu/digital-single-market/en/news/eu-cybersecurity-act-brings-strong-agency-cybersecurity-and-eu-wide-rules-cybersecurity.

92 Iain Nash, ‘Cybersecurity in a post-data environment: Considerations on the regulation of code and the role of producer and consumer liability in smart devices’ (2021) 40 Computer Law & Security Review page 3.

93 https://ec.europa.eu/digital-single-market/en/news/eu-cybersecurity-act-brings-strong-agency-cybersecurity-and-eu-wide-rules-cybersecurity.

94 Emanuele Ventrella, ‘Privacy in emergency circumstances: data protection and the COVID-19 pandemic’, 28 September 2020, ERA Forum, 379–393, 391.

95 https://ico.org.uk/about-the-ico/news-and-events/news-and-blogs/2018/01/carphone-warehouse-fined-400-000-after-serious-failures-placed-customer-and-employee-data-at-risk/.

96 https://ico.org.uk/action-weve-taken/enforcement/dsg-retail-ltd/.

97 https://ico.org.uk/about-the-ico/news-and-events/news-and-blogs/2020/03/international-airline-fined-500-000-for-failing-to-secure-its-customers-personal-data/.

98 https://ico.org.uk/action-weve-taken/enforcement/british-airways/.

99 https://edpb.europa.eu/news/national-news/2021/norwegian-dpa-issues-reprimand-telenor-inadequate-protection-personal-data_en.

100 https://ec.europa.eu/digital-single-market/en/directive-security-network-and-information-systems-nis-directive.

101 https://ec.europa.eu/digital-single-market/en/directive-security-network-and-information-systems-nis-directive.

102 Directive (EU) 2016/1148 of the European Parliament and of the Council of 6 July 2016 concerning measures for a high common level of security of network and information systems across the Union.

103 See above note, articles 14(1) and 16(1).

104 ENISA, Technical guidelines for the implementation of minimum security measures for DSPs, December 2016, available at: https://www.enisa.europa.eu/publications/minimum-security-measures-for-digitalservice-providers.

105 ENISA, Technical guidelines for the implementation of minimum security measures for DSPs, December 2016, available at: https://www.enisa.europa.eu/publications/minimum-security-measures-for-digitalservice-providers, page 41–42.

106 Johan David Michels and Ian Walden, ‘How Safe is Safe Enough? Improving Cybersecurity in Europe’s Critical Infrastructure Under the NIS Directive’ (2018) *Queen Mary School of Law Legal Studies Research Paper No. 291/2018*, page 26.

107 Phillippe Jougleux, Tatiana-Eleni Synodinou & Lilian Mitrou, Criminalisation of Criminal Attacks Against Information Systems in The Legal Regulation of Cyber Attacks (2nd ed, 2020, Wolter Kluwer) by Ioannia Iglezakis (ed), pg. 57.

108 Proposal for directive on measures for high common level of cybersecurity across the Union, repealing Directive (EU) 2016/1148, Explanatory Memorandum, page 1.

109 Proposal for directive on measures for high common level of cybersecurity across the Union, repealing Directive (EU) 2016/1148, Explanatory Memorandum, page 1.

110 Proposal for directive on measures for high common level of cybersecurity across the Union, repealing Directive (EU) 2016/1148, article 18(1).

111 Proposal for directive on measures for high common level of cybersecurity across the Union, repealing Directive (EU) 2016/1148, article 18(2)(e).

112 Proposal for directive on measures for high common level of cybersecurity across the Union, repealing Directive (EU) 2016/1148, para (30) and article 6(2).

113 https://uitspraken.rechtspraak.nl/inziendocument?id=ECLI:NL:RBDHA:2018:6310.

114 https://uitspraken.rechtspraak.nl/inziendocument?id=ECLI:NL:RBDHA:2018:6310, para 2.5 and 2.7.

115 https://uitspraken.rechtspraak.nl/inziendocument?id=ECLI:NL:RBDHA:2018:6310 para 2.16.

116 Para 3.1.

117 The Burgerlijk Wetboek (or BW).

118 Para 4.13.

119 Para 3.1.

120 Para 4.9.

121 Para 4.12.

122 Para 4.14.

123 Para 4.15.

124 Para 4.15.

125 Para 4.16.

126 Para 4.18.

127 Para 4.20.

128 Wetter, J. and Ringland, N. (December 17, 2021) Understanding the Impact of Apache Log4j Vulnerability, Google Security Blog. https://security.googleblog.com/2021/12/understanding-impact-of-apache-log4j.html.

